# Clonal hematopoiesis: elements associated with clonal expansion and diseases

**DOI:** 10.1007/s44313-025-00065-7

**Published:** 2025-03-13

**Authors:** Gangpyo Ryu, Youngil Koh, Siddhartha Jaiswal, Sung-soo Yoon

**Affiliations:** 1 Cancer Research Institute, Seoul National University, Seoul, Korea; 2 The Interdisciplinary Program of Cancer Biology, Seoul National University, Seoul, Korea; 3 Department of Internal Medicine, Seoul National University Hospital, Seoul, Korea; 4 Department of Pathology, Stanford University, Stanford, CA USA; 5 Stanford Cardiovascular Institute, Stanford University, Stanford, CA USA; 6 Institute for Stem Cell Biology & Regenerative Medicine, Stanford University, Stanford, CA USA; 7 Stanford Cancer Institute, Stanford University, Stanford, CA USA; 8 College of Medicine, Seoul National University, Seoul, Korea

**Keywords:** Clonal hematopoiesis, Inflammatory pathways, Clonal expansion, Genetic mutations

## Abstract

Clonal hematopoiesis (CH), characterized by the expansion of hematopoietic stem and progenitor cells harboring somatic mutations, has emerged as a significant age-related phenomenon with profound implications for human health. While initially recognized in the 1960s, recent technological advances have revealed its complex nature and widespread prevalence, affecting up to 84% of individuals aged ≥ 70 years. The clinical significance of CH extends beyond its well-established role as a precursor to hematological malignancies, encompassing its association with cardiovascular diseases, chronic kidney disease, and other non-malignant disorders. This comprehensive review synthesizes the current understanding of CH, focusing on recent advances in genetic and molecular mechanisms, particularly the roles of commonly mutated genes such as DNMT3A, TET2, and ASXL1. We address the emerging distinction between myeloid and lymphoid CH, their differential impacts on disease progression, and the complex interplay between CH and inflammation. Special attention is given to newly identified genetic determinants of clonal expansion rates and their implications for disease progression. The review also examines the revolutionary concept of passenger-approximated clonal expansion rate and its utility in understanding CH dynamics. Furthermore, we discuss therapeutic strategies targeting inflammatory pathways and their potential in mitigating CH-associated complications. By integrating recent findings from genetic, molecular, and clinical studies, this review provides a framework for understanding CH as a systemic condition and highlights promising directions for therapeutic interventions.

## Introduction to Clonal Hematopoiesis (CH)

### General idea of CH

In CH, hematopoietic stem and progenitor cells (HSPCs) expand abnormally, leading to an imbalance in blood cell production. CH, first recognized in the 1960s, has been linked to the aging process, and its prevalence increases with age [1] [2] [3]. CH occurs because of somatic mutations in these stem cells, which cause them to expand clonally without causing hematological cancers or cytopenia [4]. Common mutations in CH involve genes such as *DNMT3A*, *TET2*, and *ASXL1*, which mostly exhibit cytosine-to-thymine transitions, a hallmark of aging [5].

CH is associated with a higher risk of hematological cancer and increased overall mortality [5]. Individuals with CH mutations are also at a higher risk of cardiovascular diseases (CVDs), such as ischemic stroke and coronary heart disease [6]. These mutations are associated with a greater red blood cell (RBC) distribution width, which further increases mortality risk [5]. Although CH is a premalignant state and a strong indicator of potential hematological cancers, it is also associated with various non-malignant disorders [3]. This combination of cancerous and non-cancerous outcomes highlights the significant impact of CH on overall health and longevity.

### Age-related Clonal Hematopoiesis of Indeterminate Potential (CHIP) and other CH-related phenomena

**Fig. 1:**
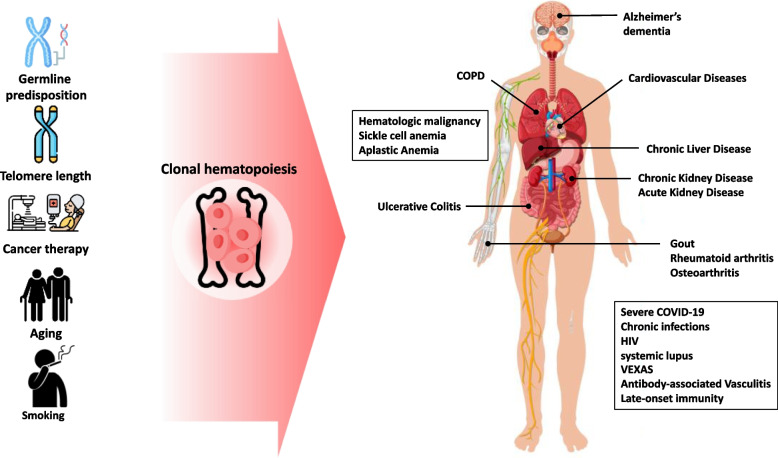
From environmental factors to diseases in CH

CHIP is a specific form of CH characterized by somatic mutations in leukemia driver genes with a variant allele fraction (VAF) of $\ge$ 2% in individuals without diagnosed blood disorders or cytopenia [3] [7]. The prevalence of CHIP increases markedly with age, ranging from 18% in individuals aged $<$ 60 years to 84% in those aged $\ge$ 70 years [8]. By the age of 70 years, approximately 20%–50% of individuals carry a CH clone, leading to a 40% increase in all-cause mortality [9]. Although CH clone size correlates with age, a recent study with longevous ($\ge$ 90 years old) and common (60–89 years old) older adult groups showed that the size of the CH clones is related to the number of mutations present rather than the person’s actual age in the longevous group [10] Fig. 1.

Other CH-related phenomena include mosaic chromosomal alterations (mCAs), which accumulate with age and are associated with hematological malignancies [3]. mCAs are age-related clonal expansions of large structural chromosomal changes in blood cells that represent a form of chromosomal mosaicism in which different karyotypes coexist within an individual originating from a single zygote [11] [12]. These structural rearrangements are detectable in a significant proportion of the older adult population and share characteristics with CH, particularly regarding their abnormal clonal expansion [13] [12].

In terms of correlation with age or abnormal clonal expansion, mCAs associated with hematological malignancy but without cytopenia and dysplastic hematopoiesis should be considered a status that falls under the CHIP category [4]. The clonal mosaic anomalies detected by Laurie et al. clustered in regions commonly associated with hematological cancers, with over 80% of these anomalies overlapping with recurrent duplications and deletions in the Mitelman Database. Despite this strong association, only 2.8% of the individuals with detectable mosaic anomalies had a prior hematological cancer diagnosis, indicating that these chromosomal changes often occur without immediate cancer diagnosis. The most common overlaps were observed in chromosomal regions such as del20q, del13q, and del11q [12]. Additionally, Loh et al. identified 8,342 mCAs in a cohort of UK Biobank participants, showing that the frequency of mCAs increased with age, similar to CHIP. Inherited genetic variants at specific loci in *MPL*, *TM2D3*-*TARSL2*, and *FRA10B* are associated with mCA acquisition and clonal selection, suggesting a genetic predisposition to CH [14].

The prevalence of mCAs is strongly influenced by age, with the frequency of clonal mosaicism increasing from < 0.5% in individuals aged < 50 years to 2%–3% in older adults [12] [15]. Other risk factors include smoking, telomere length, sex, and germline genetics [11]. In the mCAs category, myeloid mCAs (M-mCAs) are primarily linked to myeloid malignancies such as acute myeloid leukemia and myelodysplastic syndrome. These alterations often confer a competitive advantage to hematopoietic stem cells (HSCs), leading to clonal expansion and an increased risk of myeloid cancer [16] [17]. M-mCAs typically involve structural changes such as deletions or duplications that affect large chromosomal segments. These changes can disrupt normal hematopoiesis and lead to abnormal blood cell counts. They usually arise early in hematopoiesis, affecting myeloid lineage commitment and resulting in a more homogeneous population of myeloid cells [13].

In contrast, lymphoid mCAs (L-mCAs) are associated with lymphoid malignancies, including chronic lymphoblastic leukemia (CLL) and various lymphomas. Unlike M-mCAs, no significant advantages have been reported for clonal expansion in L-mCAs. L-mCAs are prevalent in older adults and can predispose individuals to lymphoid cancers [11] [17]. They often involve specific translocations and mutations in genes related to lymphoid malignancies, such as *NOTCH1* and *FBXW7* [13] [17], and can occur at various stages of lymphocyte development, including mature B and T cells, leading to a diverse array of lymphocyte clones. L-mCAs are linked to alterations in immune function and an increased risk of autoimmune disorders, in addition to lymphoid malignancies [13].

The mutation rates and fitness effects of mCAs vary significantly between different types and sexes, with the total mutation rate for highly fit mCAs being over ten-fold lower than that of highly fit single-nucleotide variants (SNVs) [18]. SNVs, indels, and copy number amplifications co-occur significantly in the same individuals, particularly affecting genes such as *DNMT3A*, *TET2*, *JAK2*, and *TP53* [19].

Multiple studies have shown that mCAs affect blood cell count and mortality rates. A strong correlation was observed between the expansion rates of *JAK2*-related mCAs and RBC count. In addition, the number and size of CH lesions were positively associated with abnormal blood counts and mortality rates from blood cancer [13] [19].

Population-level studies have provided valuable insights into mCA dynamics, with mCA fitness estimates correlating well (${R}^{2}$=0.49) with population-level clonal fraction distributions in the UK Biobank [13]. This correlation suggests that population-level data can be used to infer the fitness effects of mCAs, thereby providing a powerful tool for understanding their impact on health and disease.

In addition to mCA and CHIP, additional CH categories have been identified: CH of undetermined driver, which lacks chromosomal alterations or detectable mutations in known driver genes, and micro-CH, which involves low-abundance clones detected through high-sensitivity sequencing. Clonal cytopenia of undetermined significance, referred to as CH in the context of peripheral blood cytopenia, increases the probability of myeloid malignancy development [20]. These categories suggest a broader prevalence of CH mutations in older adults and a potential outgrowth from yet-to-be-characterized variants or genetic drift [3].

### Subtypes of CHIP: myeloid and lymphoid clonal hematopoiesis

A recent study highlighted the importance of distinguishing between myeloid CHIP (M-CHIP) and lymphoid CHIP (L-CHIP) because these two subtypes are associated with different malignancies and health outcomes. Niroula et al. demonstrated this differentiation in a study of 55,385 individuals, associating specific mutations with lymphoid malignancies and showing a higher risk of small or chronic lymphocytic lymphoma with L-CHIP [21] [22].

M-CHIP is characterized by somatic mutations in genes such as *DNMT3A*, *TET2*, and *ASXL1* and is primarily associated with an increased probability of myeloid malignancies [21]. Large M-CHIP clones are linked to an increased overall mortality rate (hazard ratio [HR] = 1.60, 95% confidence interval [CI] = 1.29–1.98, P $<$ 0.001) and coronary artery disease (CAD) risk (HR = 1.35, CI = 1.09–1.66, P = 0.005) [22].

In contrast, L-CHIP is associated with mutations in genes such as *ATM*, *KMT2D*, *DUSP22*, *FAT1*, and *SYNE1*, which can occur in HSCs and lymphoid lineage cells. L-CHIP has been identified in 1.3% of adults aged 40–70 years. The presence of L-CH influences the risk profiles of CLL, monoclonal B-cell lymphocytosis (MBL), and therapy-related lymphoid malignancies. The annual progression rate from high-count MBL to CLL ranges from 1 to 5%, whereas low-count MBL typically does not progress to CLL. L-CHIP and CLL driver mutations in MBL cells show significance [21].

Differentiation between M-CHIP and L-CHIP has been used to stratify the risks of hematopoietic malignancies, supporting the hypothesis of unique clonal hematopoietic pathways. While M-CHIP is associated with increased overall mortality rate and CAD risk, L-CHIP is specifically associated with an increased risk of lymphoid malignancies, late-onset autoimmunity, and immunodeficiency [22] [21].

### Telomere

Telomeres play crucial roles in chromosomal stability and protection, and their length is closely associated with aging and cellular division [23]. Telomere shortening is linked to aging and genomic instability and is a well-characterized cellular aging mechanism that can lead to age-related diseases [23] [24].

CH is associated with aging and cellular senescence. In dividing cells such as HSCs, reverse transcription by telomerase mitigates telomere attrition. However, this protective mechanism wanes with age, leading to the accumulation of senescent cells with pro-inflammatory phenotypes that contribute to CVD [23]. This relationship highlights the complex interplay among telomere biology, CH, and age-related diseases.

Meta-analyses have demonstrated that CHIP with a higher VAF is strongly correlated with shorter measured leukocyte telomere length (mLTL) [23]. Short leukocyte telomere lengths (LTLs) are associated with cellular senescence and genomic instability. Individuals with short telomere syndromes, such as dyskeratosis congenita, are predisposed to CH owing to reduced telomerase function [25] [24]. Conversely, Mendelian randomization (MR) studies have shown that a longer mLTL increases the risk of developing CHIP. One-sample MR studies in the TOPMed and UK Biobank cohorts consistently demonstrated a positive causal relationship between longer LTL and CHIP acquisition. Two-sample MR studies further confirmed these findings, even after accounting for pleiotropic variants, particularly at the *TERT* locus [23].

A longer LTL allows cells to evade senescence, enabling the accumulation of somatic mutations over time, suggesting that it promotes CHIP development by accelerating mutagenesis. These results highlight a clear bidirectional relationship in which a longer LTL drives CHIP acquisition through genetic and mutational mechanisms, whereas CHIP shortens the LTL. This finding suggests that having short telomeres at baseline could lead to clones becoming exhausted or losing viability over time.

POT1 plays two key roles in telomere maintenance; it shields telomeres from degradation by exonucleases and regulates telomere lengthening. Certain mutations in the *POT1* gene that result in elongated telomeres have been linked to increased susceptibility to a hereditary form of CH. This condition is associated with various non-cancerous and cancerous solid tumors [24].

Patients with telomere biology disorders (TBDs) show a distinct pattern of CH mutations compared with those without TBDs. Specifically, patients with TBDs exhibit more frequent somatic mutations in genes involved in DNA repair. These patients also have a higher incidence of CH, which is a genetic change in blood-forming cells. The increased prevalence of CH in patients with TBDs is associated with a higher risk of developing myeloid neoplasms [26]. Additionally, studies have shown that a shorter LTL is associated with CAD, whereas the effects of a longer LTL on cancer risk are mixed. Shorter mLTLs have been consistently associated with increased CH prevalence and CAD incidence. The evidence suggests a modest mediation effect of mLTL between CHIP and CAD, with mediation effects estimated at 3.4% in the UK Biobank cohort and 6.4% in the WHI cohort [23].

## Genetic and molecular aspects of CH

### Mutations and Genes

The most commonly mutated genes in CH are *DNMT3A*, *TET2*, and *ASXL1* [5] [4]. Mouse studies have shown that these genes are involved in epigenetic regulation and play crucial roles in HSC function based on mouse studies. *Dnmt3a* mutations lead to long-term HSC expansion and competitive advantage, particularly under chronic infection conditions. *Tet2* mutations result in resistance to apoptosis and clonal expansion in response to inflammation. *Asxl1* mutations are associated with reduced HSC numbers and competitive disadvantages; however, aged clones acquire growth advantages through the Akt/mTOR pathway [27]. Whether the same or different mechanisms are present in human HSCs harboring these mutations remains unknown.

**Fig. 2:**
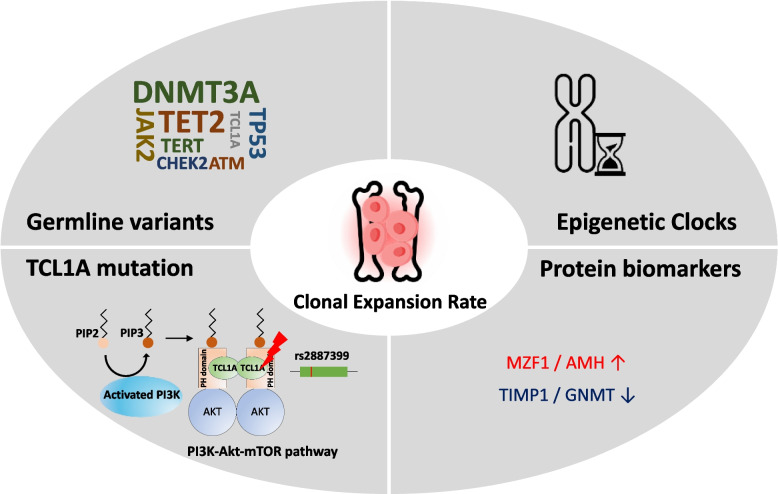
Elements affecting expansion rate in CH

Recent studies have significantly expanded our understanding of the genetic landscape in CH. Nearly 70 genes with positive selection signals were identified in CH [28]. Beauchamp et al. claimed that gene sets are associated with CH and MDS in multiple ways. Furthermore, Bernstein et al. validated and identified an additional set of genes under positive selection in CH, including *SRSF1*, *MYD88*, *CHEK2*, *ZNF234*, *ZNF318*, *IGLL5*, *MAGEC3*, *MTA2*, *YLPM1*, *BAX*, *CCL22*, *CCDC115*, *SIK3*, *SRCAP*, *SH2B3*, *SPRED2*, and *ZBTB33*. These newly discovered fitness-inferred driver genes are associated with an increased probability of hematological malignancies and various adverse health outcomes, including death, infections, and chronic obstructive pulmonary disease [29] [30] Fig. 2.

### Mechanisms of clonal expansion in CH

Aging is a primary risk factor for CH, with a significant increase in prevalence among individuals aged > 70 years. Studies have shown that 20%–50% of individuals aged ≥ 70 years carry a CH clone, leading to a 40% increase in all-cause mortality [9]. The prevalence of CHIP increases dramatically with age, from 18% in individuals aged $\le$ 60 years to 84% in those aged $\ge$ 70 years [8]. Somatic mutations in HSPCs can occur early in life, and their accumulation serves as a molecular clock for HSC dynamics [3]. Hematopoietic stressors, including chemotherapy, radiation, and inflammation, are crucial for the development and progression of CH into hematological malignancies [20].

Cancer therapies, particularly chemotherapy and radiation, are significant risk factors for the development of CH. These treatments induce mutational stress in HSCs, thereby influencing the evolution and selection of CH mutations. Specifically, mutations in DNA damage response genes (*TP53*, *PPM1D*, and *CHEK2*) are induced by radiation and certain chemotherapies [31]. CH is prevalent in heavily pretreated patients undergoing autologous hematopoietic cell transplantation [32]. These findings challenge the traditional view that therapy-related myeloid neoplasms arise solely from the mutagenic effects of cancer therapy, suggesting that some initiating mutations predate therapy [31].

Smoking is another significant risk factor for CH in a dose-dependent manner [33]. Certain mutations, particularly in *ASXL1*, are enriched in smokers [31].

Environmental exposure may contribute to CH development, as evidenced by the increased incidence of CH in firefighters exposed to the World Trade Center disaster [27]. Obesity is strongly associated with CHIP, and the accompanying pro-inflammatory state may accelerate progression to more severe hematological neoplasia [34]. The environment within the bone marrow is crucial for fostering CH. In particular, inflammatory conditions in this setting, which are shaped by diverse signaling molecules and factors outside the cell, play a significant role in the emergence and advancement of CH [35]. This underscores the complex interplay between the intrinsic genetic and extrinsic environmental factors involved in CH pathogenesis.

Genetic studies have revealed ancestry-based variations in CH susceptibility, with mutations being more common in individuals of European ancestry than in those of non-European ancestry [16]. Specifically, self-reported Hispanic or Latin ethnicities were associated with a lower prevalence of CHIP (odds ratio: 0.82, 95% CI: 0.72–0.95) [36]. Sex differences in CH mutations have been observed, with males more commonly exhibiting mutations in splicing factors and *ASXL1* [3]. Additionally, men have mosaic loss of the Y chromosome, whereas women may have higher rates of certain autosomal mCAs in addition to mosaic loss of the X chromosome [16].

Some studies have suggested that certain CH mutations have protective effects on longevity. Individuals who lived until an exceptionally old age showed a greater occurrence of CH and more frequent mutations in *TET2* and *ASXL1*, suggesting that certain types of CH play a role in extending the lifespan [10] [37].

### Positive selection and clonal expansion

Positive selection, rather than genetic drift, has been identified as the primary factor driving the evolution of CH. The fitness landscape of the CH-associated genes varies considerably, with *DNMT3A* exhibiting a broad spectrum of effects [38].

The timing of mutation acquisition and fitness conferred by these mutations play crucial roles in determining their prevalence within the hematopoietic system. Variants with high fitness acquired early in the process tend to have high VAFs, whereas those with low fitness or late acquisition typically have low VAFs [38]. This dynamic is further complicated by context-dependent factors, such as chemotherapy, radiation exposure, smoking, and inflammation, which can significantly influence clone fitness and drive clonal dominance and outgrowth [3].

Mutations in different genes exhibit varying growth rates. For example, *SRSF2*, *PTPN11*, and *U2AF1* gene mutations showed higher growth rates than mutations in genes such as *DNMT3A*, *TET2*, and *ASXL1* [39]. These differences in growth rates contribute to the complex and heterogeneous nature of CH, highlighting the need for a nuanced understanding of the various genetic and environmental factors that shape its progression.

### Genetic Predispositions and Heritability: Clonal Expansion Rate in Passenger-Approximated Clonal Expansion Rate (PACER)

Germline genetic variants play important roles in the development and risk of CH. Specific germline predispositions have been associated with different types of CH, particularly mutations in *JAK2*, *TET2*, and *DNMT3A* [40]. Genome-wide association studies (GWAS) have identified several chromosomal loci associated with CHIP and mCAs, including *TP53*, *TERT*, *ATM*, *CHEK2*, and *TCL1A* [16] [41]. GWAS have revealed approximately 70 genetic variants associated with CH, which are enriched in genes involved in DNA damage repair, cell cycle regulation, and hematopoietic progenitor cell regulation [28].

Recent studies have investigated the clonal expansion rates and their relationships with CH and other factors. Weinstock et al. developed the PACER method to study the germline determinants of clonal expansion rate in CH. This study has led to important insights into the role of *TCL1A* in promoting clonal expansion in CHIP and mCAs. The *TCL1A* gene is implicated in the PI3K-Akt-mTOR signaling pathway, which enhances the proliferative capacity and clonal expansion of HSCs. *TCL1A* is not expressed in wild-type HSCs but is aberrantly expressed in certain CHIP mutations. Notably, the presence of the T allele at rs2887399 is associated with reduced *TCL1A* expression and slower clonal expansion [41]. A comprehensive genetic study examining the mCA growth rates provided additional evidence to support this conclusion. This study identified a genetic variant in the *TCL1A* region linked to the rate of mCA clonal expansion. Additional potentially significant variants were found in *NRIP1* and *TERT* [13].

The rate of clonal expansion is also influenced by epigenetic factors. Studies have demonstrated a significant connection between clonal expansion rates and epigenetic clocks, predicted through genetic analyses and measured directly. Epigenetic clocks such as PhenoAge, GrimAge, and HannumAge are positively associated with CHIP [42] and the clonal expansion rate [43].

Proteomic analyses have identified specific circulating proteins associated with CHIP expansion rates. Proteins such as myeloid zinc finger 1 and anti-Müllerian hormone are associated with increased CHIP expansion rates, whereas tissue inhibitors of metalloproteinase 1 and glycine N-methyltransferase are associated with decreased expansion rates [43].

## Inflammation and immune response

### Inflammatory Pathways

Pro-inflammatory signaling and cytokine production play pivotal roles in the progression of CH, creating a favorable environment for the expansion of mutant clones and contributing to the associated health risks. This process involves complex interactions between mutated HSPCs and inflammatory milieu.

Inflammatory cytokines promote the survival and expansion of *TET2*-mutated HSPCs compared with their normal counterparts. This preferential expansion is partly due to cytokine-induced anti-apoptotic pathways that specifically benefit *TET2*-mutant clones [44]. This mechanism provides a selective advantage to the mutant cells under inflammatory conditions.

The loss of *TET2* in macrophages enhances the production of pro-inflammatory cytokines, including interleukin (IL)−6 and IL-1 $\beta$, in response to various stimuli such as lipopolysaccharide or cholesterol. Similarly, DNMT3A deficiency in macrophages and mast cells increases the expression of inflammatory genes [45]. These hyper-inflammatory responses are believed to contribute significantly to the increased cardiovascular risk associated with CHIP.

Certain cytokines, particularly IL-1 $\beta$ and granulocyte–macrophage colony-stimulating factor, further stimulate the proliferation and differentiation of *TET2*-mutated HSPCs into myeloid lineages, skewing hematopoiesis toward myeloid bias, which is a characteristic feature of CH [44]. Preclinical studies have demonstrated that inflammatory cytokines, such as tumor necrosis factor $\alpha$ and IL-6, provide a growth advantage to HSPCs with *TET2* and *DNMT3A* mutations, reinforcing the selective pressure favoring mutant clones in inflammatory environments [46].

The increased risk of CH is likely linked to increased inflammation driven by mutated macrophages, partly via inflammasome activation [47]. CH is associated with systemic inflammation and increased morbidity and mortality rates [48]. It is also associated with elevated inflammation and impaired tumor suppressor function, which increase the risk of CVDs and hematological malignancies [49].

Humans with *TET2* mutations show higher levels of circulating IL-8, IL-6, and IL-1 $\beta$ [50]. *ASXL1* variant promotes a pro-inflammatory phenotype in macrophages, thereby increasing cytokine production [47]. *DNMT3A* mutations lead to increased effector function in CD4 + T and natural killer cells with altered gene expression profiles [51].

Blockade of IL-6 signaling reverses atherosclerosis in *Tet2*-deficient CH mice [52]. Anti-inflammatory therapies targeting IL-1 $\beta$ and IL-6 show potential in reducing cardiovascular risks associated with clonal hematopoiesis. Specific inhibitors of inflammasomes, such as NLRP3 and AIM2, may also effectively reduce inflammation [53].

### Bone Marrow Environment

The relationship between aging, CH, and the bone marrow niche represents a complex, interconnected system that significantly affects HSC function and overall bone health. This intricate interplay is characterized by a self-reinforcing cycle of inflammation and clonal expansion.

Aging profoundly affects the bone marrow niche, contributing to the progression of CH and altering HSC function via two primary mechanisms: inflammation and changes in mesenchymal stem cell differentiation. CH also influences the bone marrow microenvironment. As mutant clones expand, they alter the composition and function of the bone marrow niche, affecting the overall bone health and creating conditions that promote CH progression [54]. Mutant leukocytes derived from the CHIP clones create an inflammatory milieu that promotes clonal expansion and disease progression [55]. This establishes a feedback loop in which the altered bone marrow environment becomes increasingly favorable for the expansion of CH clones, perpetuating a cycle of inflammation and expansion [55] [54].

The inflammatory milieu in the bone marrow microenvironment contributes to bone loss and impairs osteoblastic differentiation. This is particularly evident in the context of specific CH-associated mutations. For instance, *Dnmt3a* has been shown to decrease bone mass by increasing osteoclastogenesis. The reduction in bone density is caused by inflammatory signaling molecules, particularly IL-20. This IL-20 is produced by macrophages carrying *Dnmt3a* mutations through a process involving the Irf3-NK-$\kappa$ B pathway [56]. This signaling not only affects bone health by altering osteoblast and osteoclast activity but also actively promotes the development and expansion of CH [35]. Bone-related disorders are also associated with CH. CH is associated with osteoporosis and osteoarthritis by contributing to inflammatory processes and altered bone remodeling [54]. Animal models have provided further evidence for this association, with *Dnmt3a* and *Tet2* knockout mice showing reduced bone volume and increased osteoclastogenesis, leading to an increased risk of osteoporosis [27].

Kin et al. have provided important insights into potential therapeutic approaches. They found that alendronate, a bisphosphonate commonly used to treat osteoporosis, and IL-20 neutralization reduced osteoclastogenesis and bone loss in *Dnmt3a* mutant models. This finding suggests that targeting specific inflammatory pathways or using established osteoporosis treatments are beneficial for managing bone-related complications of CH [56].

## Therapeutic strategies and interventions

### Targeting inflammation

Anti-inflammatory therapies have emerged as promising approaches for mitigating the cardiovascular risks and other complications associated with CH. These therapies target key inflammatory pathways thought to mediate the detrimental effects of CH on cardiovascular health and other systems.

Several studies have highlighted the potential for targeting specific inflammatory pathways. Anti-inflammatory treatments focusing on IL-1 $\beta$ and IL-6 pathways have shown promise in mitigating CH-associated cardiovascular risks [53]. Similarly, JAK2 inhibitors have been identified as potential agents for reducing the CVD risk associated with CHIP [57]. These treatments directly target the inflammatory mechanisms that are believed to link CH to increased cardiovascular risk.

One notable example is the use of canakinumab, an anti-inflammatory drug that has shown promise in reducing cardiovascular events in individuals with CH [58]. This finding provides clinical evidence supporting the inflammatory hypothesis of CH-associated cardiovascular risk and suggests a potential therapeutic strategy for managing these risks.

Specific approaches targeting inflammasomes, such as NLRP3 and AIM2 inhibitors, have also been proposed as potential therapeutic strategies [53]. These targeted approaches may offer a more precise control of the inflammatory processes associated with CH.

Combination therapy has shown promising results in various experimental models. For instance, calcium channel blockers such as nifedipine and SKF-96365, either alone or in combination with metformin, MCC950, or anakinra (an IL-1 receptor antagonist), have been shown to suppress the growth of mutant CHIP cells and partially restore normal hematopoiesis in experimental models [34]. This suggests that multitarget approaches are particularly effective in managing CH and its complications.

Genetic studies have provided additional insights into the role of inflammation in CH-associated risks. The IL6R p.Asp358Ala variant, which naturally reduces IL-6 signaling, has been found to significantly mitigate the risk of CVD among individuals with large CHIP clones [59]. This genetic evidence supports the potential efficacy of IL-6 inhibition in managing CH-associated cardiovascular risk.

However, the field is still evolving, and some findings challenge established hypotheses. For instance, some studies have questioned the role of IL-6 inhibition in reducing the risk of cardiovascular disease among CHIP carriers [60].

## Concluding Remarks

In conclusion, these findings underscore the complex and multifaceted nature of CH and its widespread implications for human health. The intricate interplay between genetic mutations, especially in key genes such as *DNMT3A*, *TET2*, and *ASXL1*, and environmental factors contributes significantly to the development and progression of CH. These mutations not only increase the risk of hematological malignancies but also substantially predispose patients to cardiovascular disease, inflammatory reactions, and several other non-malignant conditions. This review highlights how CH, once considered a benign consequence of aging, is now accepted as a relevant factor in the pathogenesis of several diseases and as a possible forerunner of the most serious hematological disorders. A growing understanding of the molecular mechanisms and related risks of CH has paved the way for the development of novel therapeutic and preventive strategies. Anti-inflammatory strategies targeting specific pathways, such as IL-1 $\beta$ and IL-6, could be valuable in decreasing the cardiovascular risk attributed to CH. In addition, personalized clinical management strategies can be developed based on specific genetic alterations in individuals and their attendant risk factors, potentially leading to improved and more tailored treatments.

As knowledge in this domain continues to rapidly evolve, several important aspects will become crucial for future research. More refined methods must be developed for risk stratification, enabling clinicians to identify those at high risk of CH more precisely and effectively and implement suitable interventions. Investigating how CH contributes to other non-hematological diseases, such as autoimmunity and solid tumors, and studying the long-term effects of CH on aging and the overall health span represent highly compelling areas for further research. As our understanding of CH improves, knowledge should be translated into practice, leading to the development of targeted treatments and monitoring strategies for optimal outcomes in patients with CH. Continued study of the complexity of CH promises to provide insights into basic aging processes, cancer development, and immune functions. This study may lead to key breakthroughs in the management of a wide array of age-associated diseases.

## Data Availability

No datasets were generated or analysed during the current study.

## References

[1] Busque L, Mio R, Mattioli J, et al. Nonrandom X-Inactivation Patterns in Normal Females: Lyonization Ratios Vary With Age. Blood. 1996;88:59–65. 8704202

[2] Busque L, Patel JP, Figueroa ME, et al. Recurrent somatic TET2 mutations in normal elderly individuals with clonal hematopoiesis. Nat Genet. 2012;44:1179–81. 10.1038/ng.2413. 23001125 10.1038/ng.2413 PMC3483435

[3] Weeks LD, Ebert BL. Causes and consequences of clonal hematopoiesis. Blood. 2023;142:2235–46. 10.1182/blood.2023022222. 37931207 10.1182/blood.2023022222 PMC10862247

[4] Steensma DP, Bejar R, Jaiswal S, et al. Clonal hematopoiesis of indeterminate potential and its distinction from myelodysplastic syndromes. Blood. 2015;126:9–16. 10.1182/blood-2015-03-631747. 25931582 10.1182/blood-2015-03-631747 PMC4624443

[5] Jaiswal S, Fontanillas P, Flannick J, et al. Age-Related Clonal Hematopoiesis Associated with Adverse Outcomes. N Engl J Med. 2014;371:2488–98. 10.1056/NEJMoa1408617. 25426837 10.1056/NEJMoa1408617 PMC4306669

[6] Jaiswal S, Natarajan P, Silver AJ, et al. Clonal Hematopoiesis and Risk of Atherosclerotic Cardiovascular Disease. N Engl J Med. 2017;377:111–21. 10.1056/NEJMoa1701719. 28636844 10.1056/NEJMoa1701719 PMC6717509

[7] Kusne Y, Xie Z, Patnaik MM. Clonal hematopoiesis: Molecular and clinical implications. Leuk Res. 2022;113: 106787. 10.1016/j.leukres.2022.106787. 35091334 10.1016/j.leukres.2022.106787

[8] Uddin MM, Zhou Y, Bick AG, et al. Longitudinal profiling of clonal hematopoiesis provides insight into clonal dynamics. Immunity & Ageing. 2022;19:23. 10.1186/s12979-022-00278-9. 35610705 10.1186/s12979-022-00278-9 PMC9128083

[9] Stein A, Metzeler K, Kubasch AS, et al. Clonal hematopoiesis and cardiovascular disease: deciphering interconnections. Basic Res Cardiol. 2022;117:55. 10.1007/s00395-022-00969-w. 36355225 10.1007/s00395-022-00969-w PMC9649510

[10] Wang K, Zhang W, Yi L, et al. The impact of age and number of mutations on the size of clonal hematopoiesis. Proc Natl Acad Sci. 2024;121: e2319364121. 10.1073/pnas.2319364121. 38359296 10.1073/pnas.2319364121 PMC10895265

[11] Hubbard AK, Brown DW, Machiela MJ. Clonal hematopoiesis due to mosaic chromosomal alterations: Impact on disease risk and mortality. Leuk Res. 2023;126: 107022. 10.1016/j.leukres.2023.107022. 36706615 10.1016/j.leukres.2023.107022 PMC9974917

[12] Laurie CC, Laurie CA, Rice K, et al. Detectable clonal mosaicism from birth to old age and its relationship to cancer. Nat Genet. 2012;44:642–50. 10.1038/ng.2271. 22561516 10.1038/ng.2271 PMC3366033

[13] Pershad Y, Mack T, Poisner H, et al. Determinants of mosaic chromosomal alteration fitness. Nat Commun. 2024;15:3800. 10.1038/s41467-024-48190-8. 38714703 10.1038/s41467-024-48190-8 PMC11076528

[14] Loh P-R, Genovese G, Handsaker RE, et al. Insights into clonal haematopoiesis from 8,342 mosaic chromosomal alterations. Nature. 2018;559:350–5. 10.1038/s41586-018-0321-x. 29995854 10.1038/s41586-018-0321-x PMC6054542

[15] Jacobs KB, Yeager M, Zhou W, et al. Detectable clonal mosaicism and its relationship to aging and cancer. Nat Genet. 2012;44:651–8. 10.1038/ng.2270. 22561519 10.1038/ng.2270 PMC3372921

[16] Jakubek YA, Reiner AP, Honigberg MC. Risk factors for clonal hematopoiesis of indeterminate potential and mosaic chromosomal alterations. Translational Research: The Journal of Laboratory and Clinical Medicine. 2023;255:171–80. 10.1016/j.trsl.2022.11.009. 36414227 10.1016/j.trsl.2022.11.009 PMC10135440

[17] Zekavat SM, Lin S-H, Bick AG, et al. Hematopoietic mosaic chromosomal alterations increase the risk for diverse types of infection. Nat Med. 2021;27:1012–24. 10.1038/s41591-021-01371-0. 34099924 10.1038/s41591-021-01371-0 PMC8245201

[18] Watson CJ, Blundell JR. Mutation rates and fitness consequences of mosaic chromosomal alterations in blood. Nat Genet. 2023;55:1677–85. 10.1038/s41588-023-01490-z. 37697102 10.1038/s41588-023-01490-z PMC10562253

[19] Saiki R, Momozawa Y, Nannya Y, et al. Combined landscape of single-nucleotide variants and copy number alterations in clonal hematopoiesis. Nat Med. 2021;27:1239–49. 10.1038/s41591-021-01411-9. 34239136 10.1038/s41591-021-01411-9

[20] Warren JT, Link DC. Clonal hematopoiesis and risk for hematologic malignancy. Blood. 2020;136:1599–605. 10.1182/blood.2019000991. 32736382 10.1182/blood.2019000991 PMC8209630

[21] von Beck K, von Beck T, Ferrell PB, et al. Lymphoid clonal hematopoiesis: implications for malignancy, immunity, and treatment. Blood Cancer J. 2023;13:5. 10.1038/s41408-022-00773-8. 36599826 10.1038/s41408-022-00773-8 PMC9813350

[22] Niroula A, Sekar A, Murakami MA, et al. Distinction of lymphoid and myeloid clonal hematopoiesis. Nat Med. 2021;27:1921–7. 10.1038/s41591-021-01521-4. 34663986 10.1038/s41591-021-01521-4 PMC8621497

[23] Nakao T, Bick AG, Taub MA, et al (2022) Mendelian randomization supports bidirectional causality between telomere length and clonal hematopoiesis of indeterminate potential. Science Advances 8:eabl6579. 10.1126/sciadv.abl6579 10.1126/sciadv.abl6579 PMC8986098 35385311

[24] DeBoy EA, Tassia MG, Schratz KE, et al. Familial Clonal Hematopoiesis in a Long Telomere Syndrome. N Engl J Med. 2023;388:2422–33. 10.1056/NEJMoa2300503. 37140166 10.1056/NEJMoa2300503 PMC10501156

[25] Lasho T, Patnaik MM. Adaptive and Maladaptive Clonal Hematopoiesis in Telomere Biology Disorders. Curr Hematol Malig Rep. 2024;19:35–44. 10.1007/s11899-023-00719-2. 38095828 10.1007/s11899-023-00719-2

[26] Ferrer A, Mangaonkar AA, Patnaik MM. Clonal Hematopoiesis and Myeloid Neoplasms in the Context of Telomere Biology Disorders. Curr Hematol Malig Rep. 2022;17:61–8. 10.1007/s11899-022-00662-8. 35524933 10.1007/s11899-022-00662-8 PMC9077347

[27] Nannya Y. Factors associated with clonal hematopoiesis and interaction with marrow environment. J Bone Miner Metab. 2023;41:380–7. 10.1007/s00774-022-01380-0. 36346484 10.1007/s00774-022-01380-0

[28] Pich O, Reyes-Salazar I, Gonzalez-Perez A, Lopez-Bigas N. Discovering the drivers of clonal hematopoiesis. Nat Commun. 2022;13:4267. 10.1038/s41467-022-31878-0. 35871184 10.1038/s41467-022-31878-0 PMC9308779

[29] Beauchamp EM, Leventhal M, Bernard E, et al. ZBTB33 Is Mutated in Clonal Hematopoiesis and Myelodysplastic Syndromes and Impacts RNA Splicing. Blood Cancer Discovery. 2021;2:500–17. 10.1158/2643-3230.BCD-20-0224. 34568833 10.1158/2643-3230.BCD-20-0224 PMC8462124

[30] Bernstein N, Spencer Chapman M, Nyamondo K, et al. Analysis of somatic mutations in whole blood from 200,618 individuals identifies pervasive positive selection and novel drivers of clonal hematopoiesis. Nat Genet. 2024. 10.1038/s41588-024-01755-1. 38744975 10.1038/s41588-024-01755-1 PMC11176083

[31] Bolton KL, Ptashkin RN, Gao T, et al. Cancer therapy shapes the fitness landscape of clonal hematopoiesis. Nat Genet. 2020;52:1219–26. 10.1038/s41588-020-00710-0. 33106634 10.1038/s41588-020-00710-0 PMC7891089

[32] von Bonin M, Jambor HK, Teipel R, et al. Clonal hematopoiesis and its emerging effects on cellular therapies. Leukemia. 2021;35:2752–8. 10.1038/s41375-021-01337-8. 34215849 10.1038/s41375-021-01337-8 PMC8249428

[33] Stacey SN, Zink F, Halldorsson GH, et al. Genetics and epidemiology of mutational barcode-defined clonal hematopoiesis. Nat Genet. 2023;55:2149–59. 10.1038/s41588-023-01555-z. 37932435 10.1038/s41588-023-01555-z PMC10703693

[34] Pasupuleti SK, Ramdas B, Burns SS, et al. Obesity-induced inflammation exacerbates clonal hematopoiesis. J Clin Investig. 2023;133: e163968. 10.1172/JCI163968. 37071471 10.1172/JCI163968 PMC10231999

[35] Xie X, Su M, Ren K, et al. Clonal hematopoiesis and bone marrow inflammation. Translational Research: The Journal of Laboratory and Clinical Medicine. 2023;255:159–70. 10.1016/j.trsl.2022.11.004. 36347490 10.1016/j.trsl.2022.11.004 PMC11992924

[36] Vlasschaert C, Mack T, Heimlich JB, et al. A practical approach to curate clonal hematopoiesis of indeterminate potential in human genetic data sets. Blood. 2023;141:2214–23. 10.1182/blood.2022018825. 36652671 10.1182/blood.2022018825 PMC10273159

[37] Walsh K, Raghavachari N, Kerr C, et al. Clonal Hematopoiesis Analyses in Clinical, Epidemiologic, and Genetic Aging Studies to Unravel Underlying Mechanisms of Age-Related Dysfunction in Humans. Frontiers in Aging. 2022;3: 841796. 10.3389/fragi.2022.841796. 35821803 10.3389/fragi.2022.841796 PMC9261374

[38] Watson CJ, Papula AL, Poon GYP, et al. The evolutionary dynamics and fitness landscape of clonal hematopoiesis. Science. 2020;367:1449–54. 10.1126/science.aay9333. 32217721 10.1126/science.aay9333

[39] Fabre MA, de Almeida JG, Fiorillo E, et al. The longitudinal dynamics and natural history of clonal haematopoiesis. Nature. 2022;606:335–42. 10.1038/s41586-022-04785-z. 35650444 10.1038/s41586-022-04785-z PMC9177423

[40] Kubota Y, Viny AD. Germline predisposition for clonal hematopoiesis. Semin Hematol. 2024;61:61–7. 10.1053/j.seminhematol.2024.01.007. 38311514 10.1053/j.seminhematol.2024.01.007 PMC11103258

[41] Weinstock JS, Gopakumar J, Burugula BB, et al. Aberrant activation of TCL1A promotes stem cell expansion in clonal haematopoiesis. Nature. 2023;616:755–63. 10.1038/s41586-023-05806-1. 37046083 10.1038/s41586-023-05806-1 PMC10360040

[42] Nachun D, Lu AT, Bick AG, et al. Clonal hematopoiesis associated with epigenetic aging and clinical outcomes. Aging Cell. 2021;20: e13366. 10.1111/acel.13366. 34050697 10.1111/acel.13366 PMC8208788

[43] Mack TM, Raddatz MA, Pershad Y, et al (2024) Epigenetic and proteomic signatures associate with clonal hematopoiesis expansion rate. Nature Aging 1–10. 10.1038/s43587-024-00647-7 10.1038/s43587-024-00647-7 PMC11832052 38834882

[44] Chavakis T, Wielockx B, Hajishengallis G. Inflammatory Modulation of Hematopoiesis: Linking Trained Immunity and Clonal Hematopoiesis with Chronic Disorders. Annu Rev Physiol. 2022;84:183–207. 10.1146/annurev-physiol-052521-013627. 34614373 10.1146/annurev-physiol-052521-013627

[45] Jaiswal S, Ebert BL (2019) Clonal hematopoiesis in human aging and disease. Science 366:eaan4673. 10.1126/science.aan4673 10.1126/science.aan4673 PMC8050831 31672865

[46] Nathan DI, Dougherty M, Bhatta M, et al. Clonal hematopoiesis and inflammation: A review of mechanisms and clinical implications. Crit Rev Oncol Hematol. 2023;192: 104187. 10.1016/j.critrevonc.2023.104187. 37879493 10.1016/j.critrevonc.2023.104187

[47] Lin AE, Rauch PJ, Jaiswal S, Ebert BL. Clonal Hematopoiesis: Confluence of Malignant and Nonmalignant Diseases. Annual Review of Cancer Biology. 2022;6:187–200. 10.1146/annurev-cancerbio-060121-120026.

[48] Buttigieg MM, Rauh MJ. Clonal Hematopoiesis: Updates and Implications at the Solid Tumor-Immune Interface. JCO Precis Oncol. 2023;7: e2300132. 10.1200/PO.23.00132. 37343201 10.1200/PO.23.00132 PMC10309572

[49] Kaner J, Desai P, Mencia-Trinchant N, et al. Clonal Hematopoiesis and Premalignant Diseases. Cold Spring Harb Perspect Med. 2020;10: a035675. 10.1101/cshperspect.a035675. 31615870 10.1101/cshperspect.a035675 PMC7117948

[50] Jaiswal S. Clonal hematopoiesis and nonhematologic disorders. Blood. 2020;136:1606–14. 10.1182/blood.2019000989. 32736379 10.1182/blood.2019000989 PMC8209629

[51] Abplanalp WT, Schuhmacher B, Cremer S, et al. Cell-intrinsic effects of clonal hematopoiesis in heart failure. Nature Cardiovascular Research. 2023;2:819–34. 10.1038/s44161-023-00322-x. 39196061 10.1038/s44161-023-00322-x PMC11357996

[52] Liu W, Yalcinkaya M, Maestre IF, et al. Blockade of IL-6 signaling alleviates atherosclerosis in Tet2-deficient clonal hematopoiesis. Nature Cardiovascular Research. 2023;2:572–86. 10.1038/s44161-023-00281-3. 37539077 10.1038/s44161-023-00281-3 PMC10399458

[53] Tall AR, Fuster JJ. Clonal hematopoiesis in cardiovascular disease and therapeutic implications. Nature Cardiovascular Research. 2022;1:116–24. 10.1038/s44161-021-00015-3. 36337911 10.1038/s44161-021-00015-3 PMC9631799

[54] Winter S, Götze KS, Hecker JS, et al. Clonal hematopoiesis and its impact on the aging osteo-hematopoietic niche. Leukemia. 2024;38:936–46. 10.1038/s41375-024-02226-6. 38514772 10.1038/s41375-024-02226-6 PMC11073997

[55] Cook EK, Luo M, Rauh MJ. Clonal hematopoiesis and inflammation: Partners in leukemogenesis and comorbidity. Exp Hematol. 2020;83:85–94. 10.1016/j.exphem.2020.01.011. 32001341 10.1016/j.exphem.2020.01.011

[56] Kim PG, Niroula A, Shkolnik V, et al. Dnmt3a-mutated clonal hematopoiesis promotes osteoporosis. J Exp Med. 2021;218: e20211872. 10.1084/jem.20211872. 34698806 10.1084/jem.20211872 PMC8552148

[57] Saadatagah S, Ballantyne CM. Clonal hematopoiesis of indeterminate potential and cardiovascular disease. Translational Research: The Journal of Laboratory and Clinical Medicine. 2023;255:152–8. 10.1016/j.trsl.2022.08.013. 36067904 10.1016/j.trsl.2022.08.013 PMC12278166

[58] Polizio AH, Park E, Walsh K. Clonal Hematopoiesis: Connecting Aging and Inflammation in Atherosclerosis. Curr Atheroscler Rep. 2023;25:105–11. 10.1007/s11883-023-01083-5. 36808603 10.1007/s11883-023-01083-5 PMC10552081

[59] Bick AG, Pirruccello JP, Griffin GK, et al. Genetic Interleukin 6 Signaling Deficiency Attenuates Cardiovascular Risk in Clonal Hematopoiesis. Circulation. 2020;141:124–31. 10.1161/CIRCULATIONAHA.119.044362. 31707836 10.1161/CIRCULATIONAHA.119.044362 PMC7008855

[60] Kessler MD, Damask A, O’Keeffe S, et al. Common and rare variant associations with clonal haematopoiesis phenotypes. Nature. 2022;612:301–9. 10.1038/s41586-022-05448-9. 36450978 10.1038/s41586-022-05448-9 PMC9713173

